# Papillary Morphology, Periampullary Diverticulum, and Comorbidity Burden in Relation to Cannulation Outcomes and Procedural Difficulty in ERCP

**DOI:** 10.3390/jcm15208185

**Published:** 2026-10-21

**Authors:** Muhammed Kaya, Hüseyin Köseoğlu, Tolga Düzenli, İbrahim Durak, Mustafa Kaymazlı, Mustafa Sadeçolak

**Affiliations:** 1 Department of Gastroenterology, Hitit University Çorum Erol Olçok Training and Research Hospital, 19040 Çorum, Türkiye; 2 Department of Gastroenterology, Faculty of Medicine, Samsun University, 55080 Samsun, Türkiye; 3 Department of Gastroenterology, Faculty of Medicine, Hitit University, 19040 Çorum, Türkiye

**Keywords:** ERCP, papilla morphology, periampullary diverticulum, difficult cannulation, post-ERCP pancreatitis, Charlson Comorbidity Index

## Abstract

**Background:** Although it has been suggested that papillary morphology and periampullary diverticulum (PAD) may affect the outcomes of endoscopic retrograde cholangiopancreatography (ERCP), the independent effects of these factors have not been clearly established. In this study, we aimed to evaluate the association of papillary morphology and PAD with ERCP success, procedural difficulty, and post-ERCP complications. **Methods:** Records of 1046 ERCP procedures performed at a single center were retrospectively reviewed. The primary analysis included 693 patients with native papillae and no prior sphincterotomy. Papilla morphology was available for 381 patients and periampullary diverticulum (PAD) status for 633. Outcomes included cannulation success, difficult cannulation, cannulation time, number of cannulation attempts, post-ERCP complications, and length of hospital stay. Multivariable Firth penalized logistic regression was used to evaluate independent associations with difficult cannulation, overall complications, cannulation failure, and post-ERCP pancreatitis (PEP), adjusting for papilla morphology, PAD status, age, sex, American Society of Anesthesiologists (ASA) physical status class, and Charlson Comorbidity Index (CCI). **Results:** Regular papilla morphology, compared with the pooled group of atypical papilla morphologies (Small, Bulging, Ridged), was associated with significantly higher cannulation success (97.4% vs. 91.2%; *p* = 0.012), shorter cannulation time (median 3.0 vs. 4.0 min; *p* = 0.009), fewer cannulation attempts (*p* = 0.007), and shorter hospital stay (median 5.0 vs. 6.0 days; *p* = 0.013). The presence of a PAD was not significantly associated with any procedural outcome or complication (all *p* > 0.05). CCI was independently associated with cannulation failure (adjusted OR 1.40 per one-point increase; 95% CI 1.03–1.91; *p* = 0.031) and was the only factor reaching statistical significance across the four multivariable models. The multivariable model showed modest discrimination for difficult cannulation (area under the receiver operating characteristic curve [AUC] = 0.596; 95% CI 0.515–0.675). **Conclusions:** Atypical papillary morphology was associated with lower cannulation success, longer cannulation time, and more cannulation attempts in univariable analyses; however, neither papillary morphology nor periampullary diverticulum was independently associated with ERCP outcomes. Among the variables evaluated, higher CCI was the only factor independently associated with cannulation failure; however, this finding should be interpreted cautiously given the small number of failure events.

## 1. Introduction

Endoscopic retrograde cholangiopancreatography (ERCP) is the primary therapeutic method for treating biliary and pancreatic duct diseases [1]. However, successful deep biliary cannulation, a technical prerequisite for most therapeutic interventions, is not always easily achieved [2,3]. Even in the practice of experienced endoscopists, difficult cannulation is relatively common and is associated with an increased risk of post-ERCP pancreatitis (PEP), the most common major adverse event after ERCP [4]. In unselected populations, the incidence of PEP has been reported to be approximately 9.7% [5]. The European Society for Gastrointestinal Endoscopy (ESGE) defines difficult biliary cannulation as more than five contacts with the papilla, cannulation attempts lasting longer than five minutes after visualization of the papilla, or multiple inadvertent pancreatic duct cannulations or contrast injections [6,7]. Difficulty in cannulation increases both the risk of technical failure and PEP [8]. Therefore, identifying the anatomical and patient-specific factors associated with difficult cannulation can be clinically important for procedure planning and risk assessment.

The macroscopic morphology of the major duodenal papilla is one of the potential factors that may affect cannulation difficulty [9]. The Haraldsson classification, the first classification system to evaluate inter-observer and intra-observer agreement regarding papilla morphology, divides papillae into four groups: regular (Type I), small (Type II), protruded or pendulated (Type III), and ridged (Type IV) [10]. A systematic review and meta-analysis published in 2024 reported that difficult cannulation rates varied according to papilla morphology; this rate was 26% in regular papillae, 39% in small papillae, and 41% in convoluted or protruding papillae; however, the difference between Haraldsson categories did not reach statistical significance (*p* = 0.075) [11]. Certain papillary morphologies, particularly small papillae, have also been associated with a higher frequency of PEP. Despite these findings, papillary morphology is not routinely included in ERCP risk assessment, and its independent contribution to procedural outcomes has not yet been fully established [9,12].

Periampullary diverticulum (PAD) has traditionally been considered a potential anatomical factor that may complicate biliary cannulation, although current evidence is inconsistent on this matter. In a meta-analysis including twenty-six studies, PAD was reported to be associated with increased cannulation failure when all studies were considered together [13]. However, when the analysis was limited to studies conducted after 2000, this relationship was no longer found to be statistically significant; this suggests that advances in ERCP techniques may have reduced the impact of PAD on cannulation success [13]. In contrast, intradiverticular papillae remained associated with a higher risk of cannulation failure. The same meta-analysis also reported associations between PAD and PEP, perforation, and bleeding [13]. These findings suggest that the relationship between PAD and ERCP outcomes may be too complex to be explained solely by the presence or absence of a diverticulum, and may vary depending on anatomical features and procedural conditions.

Patient comorbidity burden is one of the patient-related factors that can affect ERCP outcomes. However, risk assessments for ERCP have primarily focused on anatomical and procedural factors, while the role of standardized comorbidity measures such as the Charlson Comorbidity Index (CCI) has been examined to a more limited extent [14,15]. Previous studies have shown that an increased burden of comorbidities may be associated with adverse events and mortality after ERCP [16,17]. In contrast, the direct relationship between the burden of comorbidities and procedural outcomes, particularly the success or failure of biliary cannulation, has not been sufficiently clarified.

To the best of our knowledge, studies evaluating papillary morphology and PAD alongside comorbidity burden within the same analytical framework are limited [12,18]. Methodologically, restricting the analysis to patients with native papillae is important because prior sphincterotomy alters papillary anatomy and may affect both the reliable assessment of papillary morphology and the characteristics of subsequent biliary cannulation [19]. Therefore, we aimed to evaluate the associations of papillary morphology and PAD with cannulation success, procedural difficulty, and post-ERCP complications in patients with native papillae, while accounting for age, sex, American Society of Anesthesiologists (ASA) physical status class, and comorbidity burden as measured by the CCI.

## 2. Methods

### 2.1. Study Design, Setting, and Patients

This was a retrospective, single-center cohort study including consecutive ERCP procedures performed at a high-volume tertiary ERCP center between January 2021 and December 2023. All interventions were conducted by three senior endoscopists, each with a procedural volume exceeding 1000 ERCPs. The study was conducted in accordance with the Declaration of Helsinki and was approved by the Clinical Research Ethics Committee of Hitit University Faculty of Medicine (Approval no. 2024-74). Given the retrospective design, the requirement for individual informed consent was waived. The study was reported in accordance with the STROBE (Strengthening the Reporting of Observational Studies in Epidemiology) guidelines for cohort studies.

**Figure 1:**
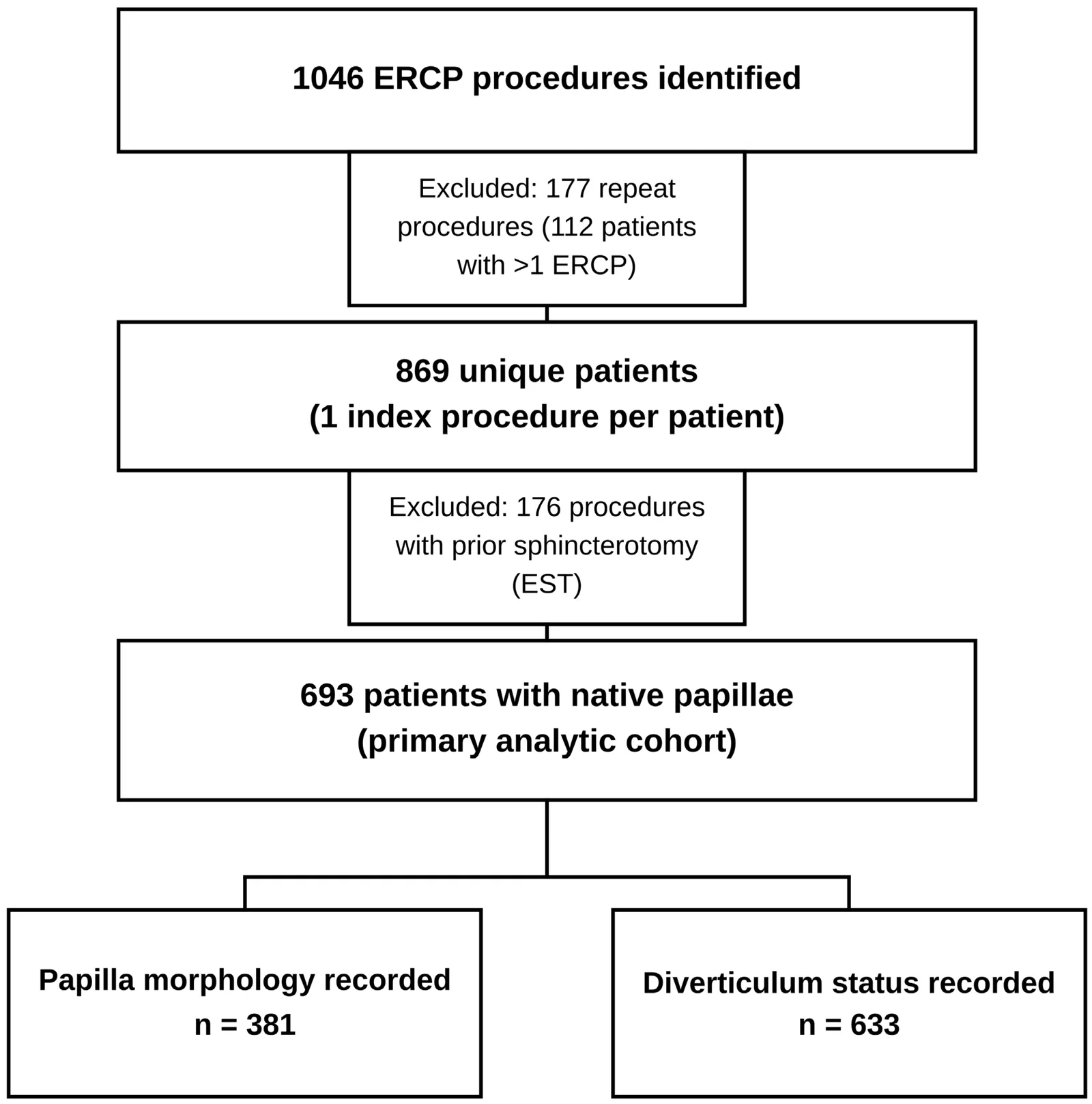
Patient flow diagram from the source database to the primary analytic cohort of patients with native papillae.

The source database included 1046 ERCP procedures. To ensure statistical independence of observations, only the first (index) ERCP procedure for each patient was included in the analysis, resulting in 869 unique patients. A total of 177 repeat procedures from 112 patients who underwent more than one ERCP during the study period were excluded. Because native papilla morphology cannot be reliably assessed in patients who have previously undergone endoscopic sphincterotomy (EST), and because prior EST substantially alters subsequent cannulation characteristics, the primary analytic cohort was restricted to patients without prior EST. Accordingly, 176 procedures in patients with documented prior EST were excluded, leaving 693 index ERCP procedures in patients with native papillae for the primary analysis (Figure 1).

### 2.2. Data Collection, Definitions, and Data Quality

**Figure 2:**
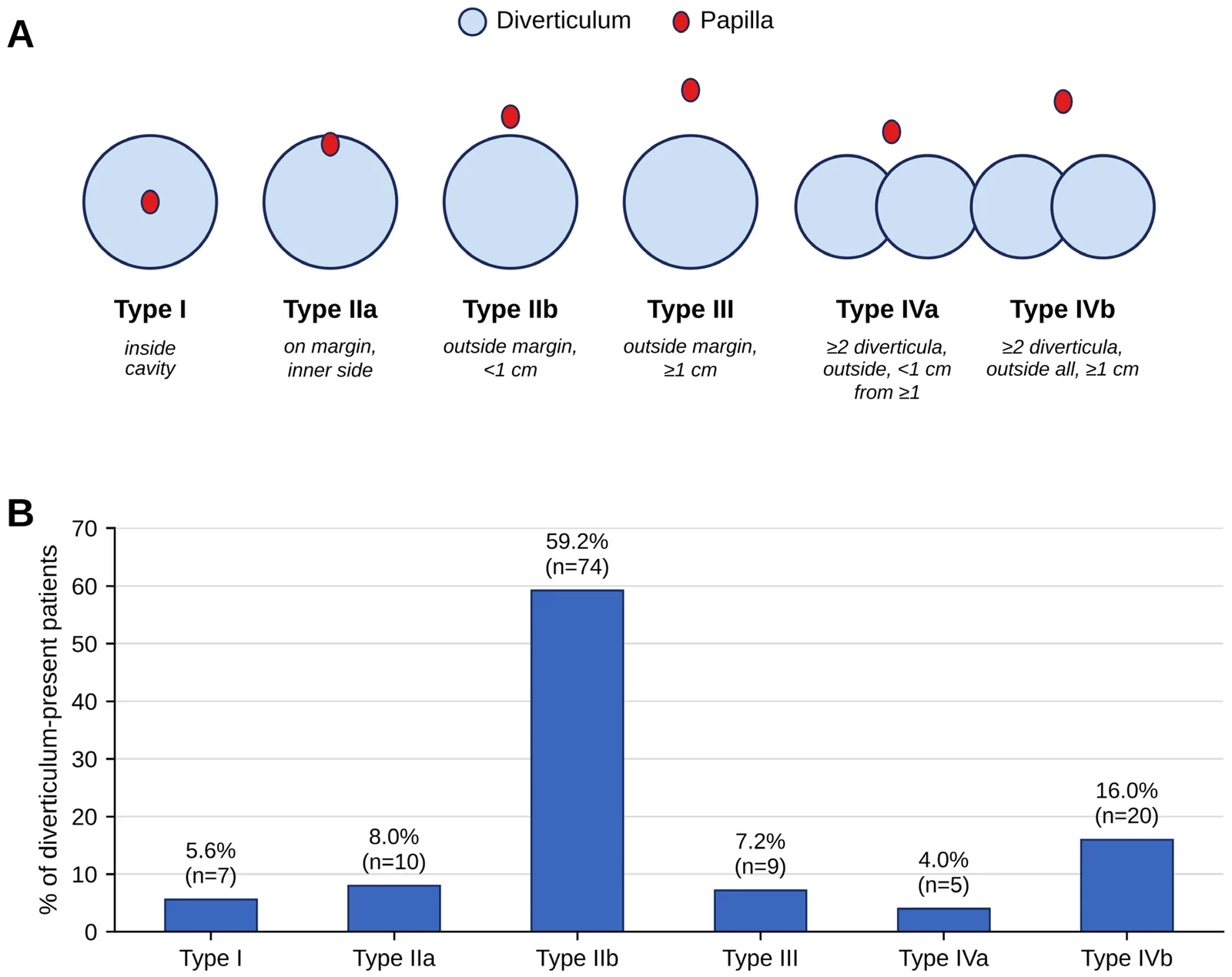
Li–Tanaka classification of periampullary diverticulum and subtype distribution in the study cohort. (**A**) Schematic representation of the Li–Tanaka classification based on the position of the papilla relative to the diverticular margin. The schematic was created by the authors according to the classification described by Yue et al. [20]. (**B**) Distribution of Li–Tanaka subtypes among the 125 patients with periampullary diverticulum (PAD). Subtype information was available for all 125 patients.

Data were retrospectively extracted from institutional medical records and ERCP procedure reports. Papilla morphology was assessed in real time by the performing endoscopist and documented in the ERCP procedure report or the patient’s medical file at the time of the procedure; for the present study, these data were retrospectively retrieved from these records. Papilla morphology was classified into four categories: Regular, Small, Bulging, and Ridged. These categories were structurally analogous to the regular, small, protruding/pendulous, and creased/ridged papilla categories, respectively, in the validated Haraldsson classification [10]. PAD status was recorded as present or absent. In patients with PAD, the diverticulum subtype was further classified according to the Li–Tanaka classification [20]. In this classification, diverticula were categorized according to the position of the papilla relative to the diverticular margin as follows: Type I (papilla located within the diverticular cavity and not adjacent to the margin), Type IIa (papilla located on the inner side of the diverticular margin), Type IIb (papilla located on the outer side of the diverticular margin and <1 cm from the margin), Type III (papilla located outside the diverticular margin and ≥1 cm from the margin), and, in patients with two or more diverticula, Type IVa (papilla located outside the margin of at least one diverticulum and <1 cm from the margin) or Type IVb (papilla located outside the margins of all diverticula and ≥1 cm from the margins) [20] (Figure 2A). PAD subtypes were reported descriptively, and exploratory outcome comparisons were performed across subtypes.

For each procedure, cannulation success, difficult cannulation, cannulation time, and number of cannulation attempts were routinely documented in the patients’ medical records at the time of the procedure. Difficult biliary cannulation was defined according to the ESGE criteria as more than five contacts with the papilla while attempting cannulation, more than five minutes of cannulation attempts after visualization of the papilla, or more than one unintended pancreatic duct cannulation or opacification [7]. Post-ERCP complications were defined according to Cotton criteria [21]. Post-ERCP outcomes included overall complications, PEP, bleeding, perforation, cholangitis, cardiopulmonary events, and length of hospital stay. Complete follow-up data for these outcomes were not available for all patients. Therefore, analyses were performed using available-case data, with percentages calculated using the number of patients with available data for each respective outcome as the denominator. The corresponding denominators are provided in the footnotes of each table. Incomplete outcome ascertainment was considered a limitation of the study.

The cannulation technique used during the procedure was recorded as sphincterotome-assisted cannulation, standard catheter cannulation, needle-knife precut papillotomy, double-guidewire (pancreatic guidewire-assisted) technique, needle-knife fistulotomy, or transpancreatic septotomy. Sphincterotome-assisted cannulation was used as the initial technique in all procedures; if this was unsuccessful, standard catheter cannulation was attempted, followed by advanced/rescue techniques in a stepwise manner. Needle-knife precut papillotomy, the double-guidewire technique, needle-knife fistulotomy, and transpancreatic septotomy were considered advanced/rescue cannulation techniques.

Comorbidity burden was assessed using the Charlson Comorbidity Index (CCI), calculated from the comorbidities documented in the medical records according to the original weighting system [22]. The index comprises 19 conditions: myocardial infarction, congestive heart failure, peripheral vascular disease, cerebrovascular disease, dementia, chronic pulmonary disease, connective tissue disease, peptic ulcer disease, mild liver disease, and diabetes without end-organ damage (1 point each); hemiplegia, moderate or severe renal disease, diabetes with end-organ damage, any tumor without metastasis, leukemia, and lymphoma (2 points each); moderate or severe liver disease (3 points); and metastatic solid tumor and acquired immunodeficiency syndrome (AIDS) (6 points each) [22]. In the age-adjusted version used in our database, 1 point is added for each decade of age from 50 years onward [23].

### 2.3. Statistical Analysis

The normality of continuous variables was assessed using the Shapiro–Wilk test. Continuous variables were summarized as median (interquartile range [IQR]) when they did not meet the assumption of normal distribution, and the Mann–Whitney U test was used for comparisons between two groups, while the Kruskal–Wallis test was used for comparisons between more than two groups. Categorical variables were presented as percentages and compared using the chi-square test. Fisher’s exact test was used when more than 20% of the expected cell frequencies were below 5.

Papillary morphology was analyzed hierarchically. First, to assess the overall association of non-regular papillary morphology with study outcomes, patients with Regular papillae were compared with a pooled “Others” group comprising Small, Bulging, and Ridged papillae. Second, the four morphological categories were analyzed separately to explore morphology-specific differences. PAD was considered a binary variable (present/absent) in all analyses due to the low number of subgroups. Multivariable analyses of four clinical outcomes—difficult cannulation, overall post-ERCP complications, cannulation failure, and PEP—were performed using the Firth penalized logistic regression method. Four categories of papillary morphology, presence of PAD, age, sex, ASA class, and CCI were included simultaneously as covariates in the models. Because the CCI recorded in our database was age-adjusted, the age points (1 point each for the age groups 50–59, 60–69, 70–79, and ≥80 years, i.e., 1–4 points) were subtracted from the recorded score, and the resulting comorbidity-only CCI was entered into the models together with age, to avoid double counting of age and collinearity between the two variables. Multivariable analyses were conducted using complete cases with available data for all covariates included in each model; therefore, the analytic sample size varied across models. The Firth method was preferred because quasi-complete separation was observed in this dataset in the standard maximum likelihood logistic regression. This was particularly due to the low number of patients in the ridged papilla group (n = 12) and the low incidence of some complications. The deviation of some regression coefficients towards infinity in the standard estimation method prevented reliable statistical inferences. Firth’s method overcomes this problem by providing finite and more stable effect estimates and is considered an accepted approach used in logistic regression analyses of rare events and small sample sizes [24].

Because perforation and cholangitis events were too rare to allow for formal statistical analysis in the papillary morphology subgroups, these results are reported only as descriptive. The discriminative performance of the multivariable models for difficult cannulation and cannulation failure was evaluated using receiver operating characteristic (ROC) curves; the areas under the curve (AUCs) and 95% confidence intervals were calculated using 2000-replicate bootstrap resampling. Given the small number of cannulation failure events, the ROC analysis for this outcome was considered exploratory. A two-sided *p*-value < 0.05 was considered statistically significant. Statistical analyses were performed using IBM SPSS Statistics for Windows, Version 27.0 (IBM Corp., Armonk, NY, USA) and R software, Version 4.5.3 (R Foundation for Statistical Computing, Vienna, Austria).

## 3. Results

### 3.1. Patient Flow and Cohort Characteristics

Of the 1046 ERCP procedures recorded, 869 were first (index) procedures. Among these patients, 693 had native papillae without prior sphincterotomy and constituted the primary analytic cohort (Figure 1). Among the 502 patients with available age data, the median age was 70.5 years (IQR, 57.0–80.0; mean ± SD, 66.9 ± 17.0 years), and 386 of 693 patients (55.7%) were female. Among the 604 patients with available etiological data, 500 (82.8%) had a biliary etiology. Among the 594 patients with a documented indication for ERCP, the indications were benign biliary disease (mainly choledocholithiasis) in 491 (82.7%), malignant biliary obstruction in 59 (9.9%), benign biliary stricture in 29 (4.9%), postoperative bile duct injury (Strasberg classification) in 9 (1.5%), biliary stent exchange in 3 (0.5%), and hydatid cyst–related biliary disease in 3 (0.5%). Periampullary diverticulum (PAD) was present in 125 of the 633 patients (19.7%) with recorded PAD status. Papilla morphology was documented in 381 patients, with the following distribution: Regular, 61.4%; Bulging, 23.4%; Small, 12.1%; and Ridged, 3.1%.

When procedural and clinical outcomes were compared across the indications for ERCP, significant differences were observed in difficult cannulation (*p* < 0.001), use of advanced/rescue cannulation techniques (needle-knife precut, double-guidewire technique, needle-knife fistulotomy, or transpancreatic septotomy; *p* = 0.003), cannulation time (*p* < 0.001), number of cannulation attempts (*p* < 0.001), and length of hospital stay (*p* < 0.001), whereas the difference in cannulation success did not reach statistical significance (*p* = 0.079). These differences were mainly driven by malignant biliary obstruction, which was associated with the lowest cannulation success rate (86.4%), the highest rates of difficult cannulation (64.2%) and advanced/rescue technique use (39.2%), the longest median cannulation time (7.0 min), the highest number of cannulation attempts (median, 4), and a longer median hospital stay (10 days). The corresponding values were 95.1%, 30.6%, 15.2%, 3.0 min, 2, and 5 days in patients with benign biliary disease, and 89.7%, 44.4%, 11.5%, 4.0 min, 2, and 5 days in patients with benign biliary stricture. Overall complication (*p* = 0.674) and PEP (*p* = 0.813) rates did not differ significantly across indications. The postoperative bile duct injury, biliary stent exchange, and hydatid cyst subgroups comprised only 9, 3, and 3 patients, respectively, precluding meaningful interpretation of their results. Suspected sphincter of Oddi dysfunction (SOD) was documented in 15 of the 346 patients (4.3%) with available data; although cannulation was successful in all of these patients, PEP developed in 7 of the 13 (53.8%) with available follow-up data.

The cannulation technique was sphincterotome-assisted in 478 of 619 patients (77.2%) and standard catheter cannulation in 36 (5.8%), whereas advanced/rescue techniques were used in 105 patients (17.0%), comprising transpancreatic septotomy in 42 (6.8%), needle-knife precut papillotomy in 26 (4.2%), the double-guidewire technique in 24 (3.9%), and needle-knife fistulotomy in 13 (2.1%). The distribution of cannulation techniques differed according to papilla morphology (*p* = 0.002): standard catheter cannulation (7.2% vs. 3.1%), needle-knife precut papillotomy (5.8% vs. 1.3%), and needle-knife fistulotomy (5.1% vs. 0.9%) were more frequently used in the Others group, whereas transpancreatic septotomy was more frequently used in the Regular group (10.9% vs. 6.5%); however, the overall rate of advanced/rescue technique use did not differ significantly between the Regular and Others groups (15.3% vs. 21.0%; *p* = 0.161). Neither the distribution of techniques (*p* = 0.107) nor the use of advanced/rescue techniques (PAD present vs. absent: 11.7% vs. 18.1%; *p* = 0.092) differed significantly according to PAD status. Advanced/rescue techniques were used more frequently in patients with malignant biliary obstruction than in those with benign biliary disease (39.2% vs. 15.2%; *p* < 0.001), and in 48.0% of patients with difficult cannulation compared with 1.7% of those without (*p* < 0.001).

### 3.2. Baseline Characteristics and Outcomes by Periampullary Diverticulum Status

Patients with PAD were significantly older than those without PAD (median, 77.0 vs. 69.0 years; *p* = 0.0002), had a higher CCI (*p* = 0.008), and had a higher ASA physical status class (*p* = 0.030). In addition, the rate of malignant biliary stricture was lower in patients with PAD (3.6% vs. 12.1%; *p* = 0.015). No significant between-group differences were observed in sex or benign stricture rate (Table 1).

Despite these differences in baseline characteristics, PAD presence was not significantly associated with any procedural outcome or complication, including cannulation success, difficult cannulation, cannulation time, number of cannulation attempts, overall complications, PEP, bleeding, perforation, cholangitis, cardiopulmonary events, or length of hospital stay (all *p* > 0.05; Table 1).

### 3.3. Periampullary Diverticulum Subtypes

Among the 125 patients with PAD, Type IIb was the most common Li–Tanaka subtype (59.2%; n = 74), followed by Type IVb (16.0%; n = 20) (Figure 2). Types I, IIa, III, and IVa were each observed in 10 or fewer patients. No statistically significant differences were observed across the six Li–Tanaka subtypes in cannulation success, difficult cannulation, cannulation time, number of cannulation attempts, overall complications, PEP, cardiopulmonary events, length of hospital stay, or malignant or benign stricture rates (all *p* > 0.10). Numerically, the difficult cannulation rate ranged from 0% in Type IIa (n = 10) to 60.0% in Type IVa (n = 5).

### 3.4. Papilla Morphology and Procedural Outcomes

Patients with (n = 381) and without (n = 312) recorded papilla morphology were comparable with respect to age, sex, CCI, ASA class, PAD prevalence, malignant stricture rate, and the proportion with a benign biliary indication, although benign biliary stricture was more frequent among patients with recorded morphology (11.9% vs. 4.6%; *p* = 0.001). The rates of difficult cannulation (33.5% vs. 35.0%; *p* = 0.694) and advanced/rescue technique use (17.4% vs. 16.3%; *p* = 0.703) were also similar between the two groups. However, cannulation success was lower (88.8% vs. 95.0%; *p* = 0.002), and overall complication (22.5% vs. 8.5%; *p* < 0.001) and PEP (16.2% vs. 6.1%; *p* < 0.001) rates were higher among patients without recorded papilla morphology.

In the binary comparison of Regular papilla morphology versus the pooled Others group comprising Small, Bulging, and Ridged morphologies, no significant differences were observed in age, sex, CCI, or ASA physical status class. However, the Others group had a significantly higher rate of malignant biliary stricture than the Regular group (16.8% vs. 5.2%; *p* = 0.002) and a significantly lower rate of benign stricture (6.7% vs. 15.2%; *p* = 0.040) (Table 2).

Patients with Regular papilla morphology had a significantly higher cannulation success rate than those with atypical papilla morphology (97.4% vs. 91.2%; *p* = 0.012), shorter cannulation time (median, 3.0 vs. 4.0 min; *p* = 0.009), fewer cannulation attempts (median, 2.0 vs. 2.0; *p* = 0.007), and shorter hospital stay (median, 5.0 vs. 6.0 days; *p* = 0.013). Difficult cannulation was numerically less frequent in the Regular group (29.6% vs. 39.9%), although the difference did not reach statistical significance (*p* = 0.054). No significant differences were observed between the Regular and Others groups in overall complications, PEP, bleeding, or cardiopulmonary events (all *p* > 0.05) (Table 2).

When the pooled Others category was separated into the individual papilla morphology groups, significant differences were observed across the four groups in cannulation success (*p* = 0.044), cannulation time (*p* = 0.020), number of cannulation attempts (*p* = 0.005), and length of hospital stay (*p* = 0.034) (Table 3). Median cannulation time was 3.0 min (IQR, 1.0–5.0) in the Regular group and 4.0 min (IQR, 3.5–9.5) in the Ridged group, while the median number of cannulation attempts was 2.0 (IQR, 1.0–3.0) and 3.0 (IQR, 2.0–5.0), respectively. Median length of hospital stay was 5.0 days (IQR, 4.0–7.0) in the Regular group and 8.0 days (IQR, 6.0–8.5) in the Ridged group.

Difficult cannulation occurred in 29.6% of the Regular, 43.2% of the Small, 36.8% of the Bulging, and 50.0% of the Ridged groups; however, the overall difference did not reach statistical significance (*p* = 0.150). No significant differences were observed in overall complications, PEP, or bleeding (all *p* > 0.05), and no perforation or cholangitis events were observed among patients with recorded papilla morphology. A nominally significant difference was observed in cardiopulmonary events (*p* = 0.002); however, this comparison was based on only two events among 247 patients.

Malignant biliary stricture rates differed significantly across the four morphology groups (*p* = 0.004), increasing from 5.2% in the Regular group to 15.4% in the Small group, 15.7% in the Bulging group, and 30.0% in the Ridged group. In contrast, benign biliary stricture rates did not differ significantly across the four groups (*p* = 0.158). Given the small size of the Ridged subgroup (n = 12), estimates for this group should be interpreted cautiously.

### 3.5. Multivariable Analysis

**Figure 3:**
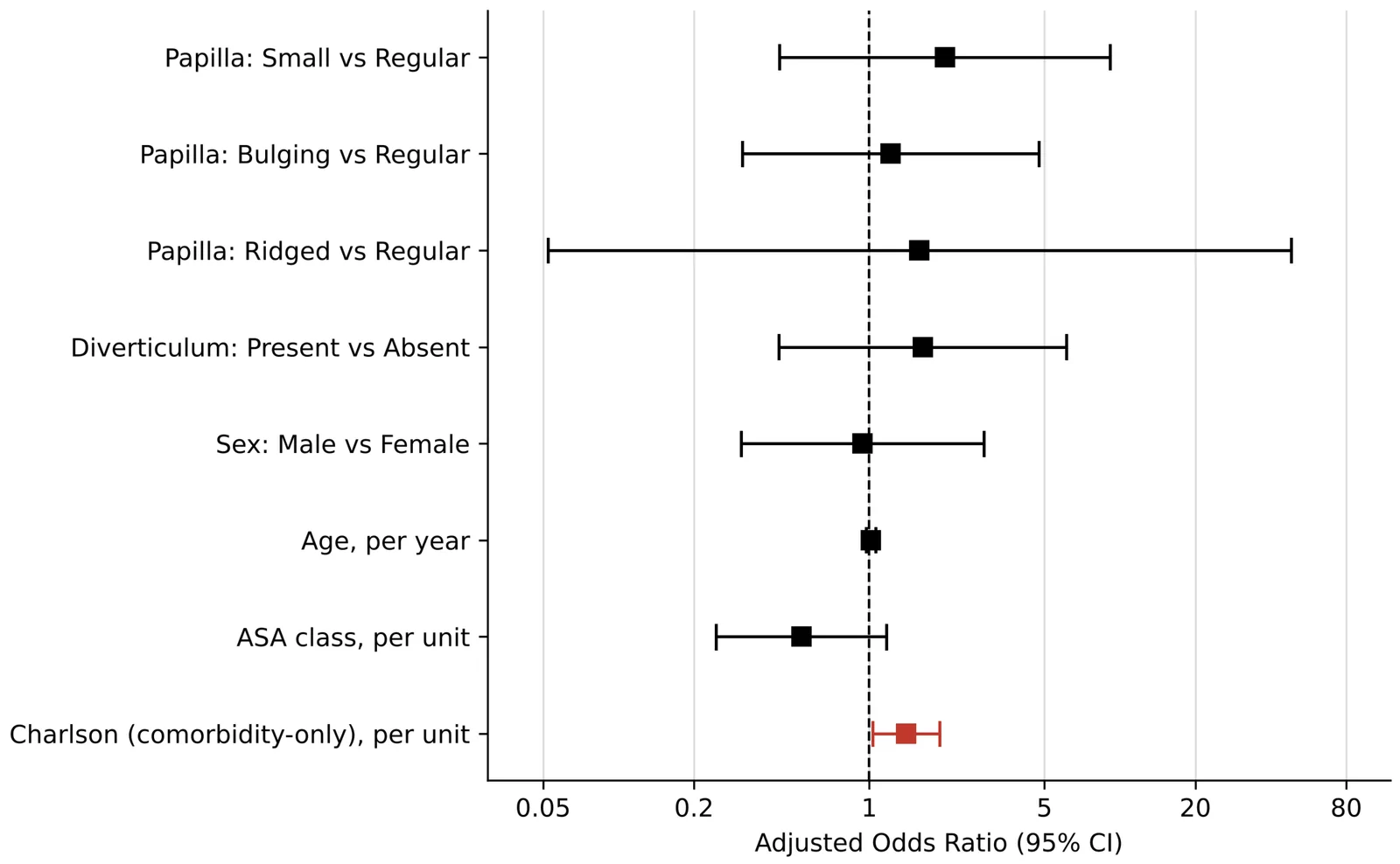
Forest plot of the multivariable Firth-penalized logistic regression model for cannulation failure. Squares represent adjusted odds ratios (ORs), and horizontal whiskers represent 95% confidence intervals (CIs). The vertical dashed line indicates OR = 1.0 (no association). The CCI, the only variable independently associated with cannulation failure at *p* < 0.05, is highlighted in red. Black squares indicate statistically non-significant associations (*p* ≥ 0.05).

Four separate multivariable Firth penalized logistic regression models were constructed, with simultaneous adjustment for papilla morphology, PAD status, age, sex, ASA physical status class, and CCI. Of these, only the cannulation failure model identified an independently significant factor (Table 4, Figure 3). The other three models—difficult cannulation, overall post-ERCP complications, and PEP—did not identify any independently significant factors.

In the multivariable model for cannulation failure, CCI was independently associated with cannulation failure, with a 40% increase in the odds of failure for each one-point increase in CCI (adjusted OR [aOR] = 1.40; 95% CI, 1.03–1.91; *p* = 0.031). CCI was the only variable that reached statistical significance across the four multivariable models. None of the other variables included in the cannulation failure model reached statistical significance (Table 4, Figure 3).

In the multivariable model for difficult cannulation (n = 215; 71 events), none of the included variables reached statistical significance. With Regular papilla morphology as the reference category, the adjusted ORs were 1.84 (95% CI, 0.76–4.46; *p* = 0.176) for Small, 1.58 (95% CI, 0.79–3.18; *p* = 0.195) for Bulging, and 1.81 (95% CI, 0.28–11.60; *p* = 0.530) for Ridged papilla morphology. PAD presence (adjusted OR = 0.85; 95% CI, 0.39–1.85; *p* = 0.686), sex (adjusted OR = 1.26; 95% CI, 0.70–2.26; *p* = 0.439), age (adjusted OR = 1.01; 95% CI, 0.99–1.03; *p* = 0.303), ASA physical status class (adjusted OR = 0.98; 95% CI, 0.71–1.35; *p* = 0.910), and CCI (adjusted OR = 1.01; 95% CI, 0.84–1.22; *p* = 0.911) were also not significantly associated with difficult cannulation.

In the multivariable model for overall post-ERCP complications (n = 213; 19 events), none of the included variables reached statistical significance. PAD presence was associated with numerically lower odds of overall complications, although the association was not statistically significant (adjusted OR = 0.09; 95% CI, 0.01–1.18; *p* = 0.067). Papilla morphology categories (adjusted OR range, 0.52–2.20; all *p* > 0.24), sex (adjusted OR = 1.24; *p* = 0.655), age (adjusted OR = 1.00; *p* = 0.895), ASA physical status class (adjusted OR = 0.59; *p* = 0.093), and CCI (adjusted OR = 1.26; *p* = 0.114) were not significantly associated with overall complications.

Similarly, in the multivariable model for PEP (n = 213; 14 events), none of the included variables reached statistical significance. PAD presence was associated with numerically lower odds of PEP, but this association was not statistically significant (adjusted OR = 0.11; 95% CI, 0.01–1.50; *p* = 0.098). No significant associations were observed for papilla morphology categories (adjusted OR range, 0.53–1.99; all *p* > 0.36), sex (adjusted OR = 1.35; *p* = 0.567), age (adjusted OR = 1.01; *p* = 0.598), ASA physical status class (adjusted OR = 0.52; *p* = 0.076), or CCI (adjusted OR = 1.09; *p* = 0.658).

### 3.6. Discriminative Performance of the Multivariable Models

**Figure 4:**
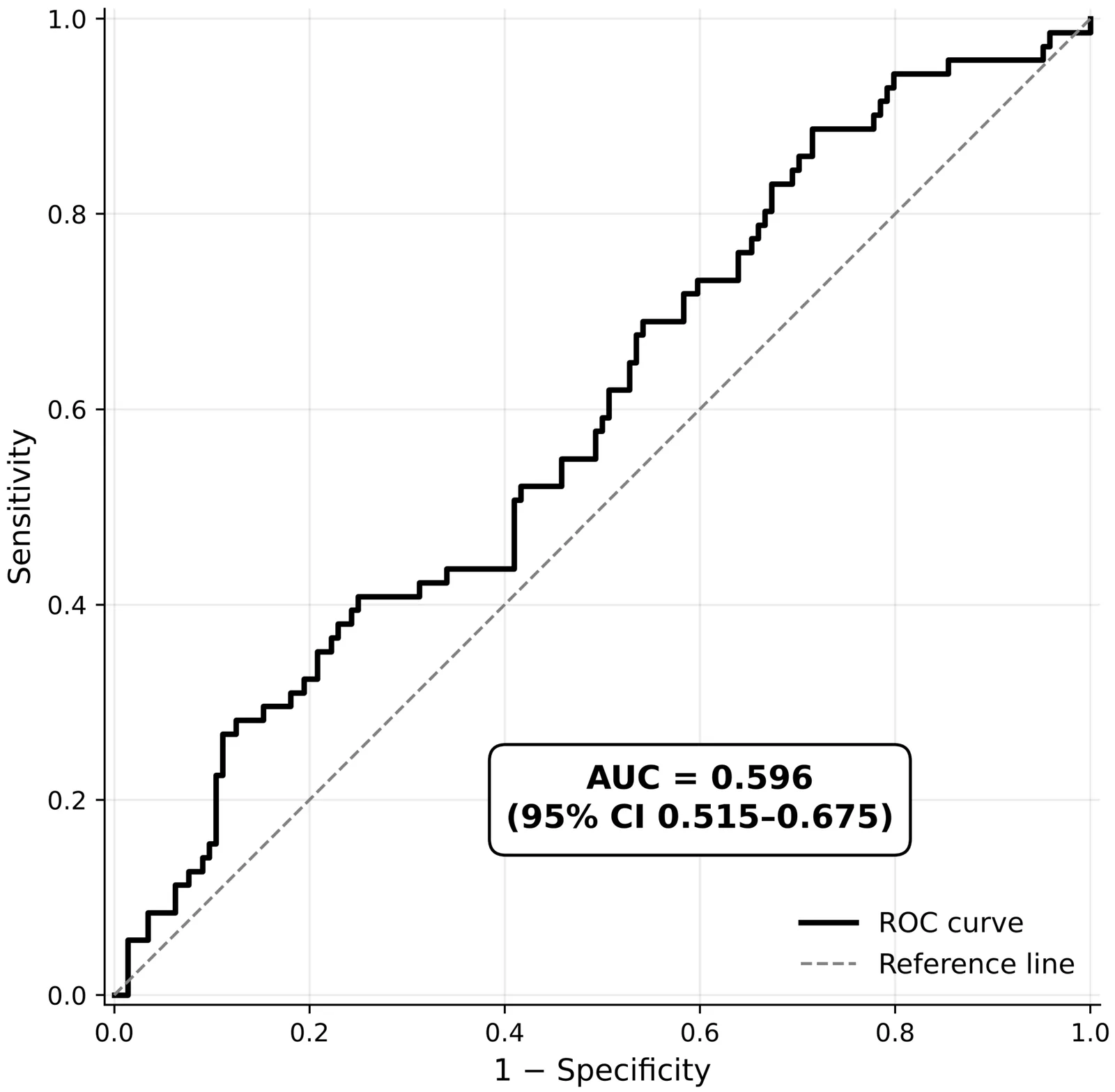
ROC Curve for the Multivariable Model of Difficult Cannulation.

To evaluate the discriminative performance of the combined predictors, a ROC curve was generated using the predicted probabilities from the Firth-penalized logistic regression model for difficult cannulation (n = 215; 71 events). The model showed an AUC of 0.596 (95% bootstrap CI, 0.515–0.675) (Figure 4).

**Figure 5:**
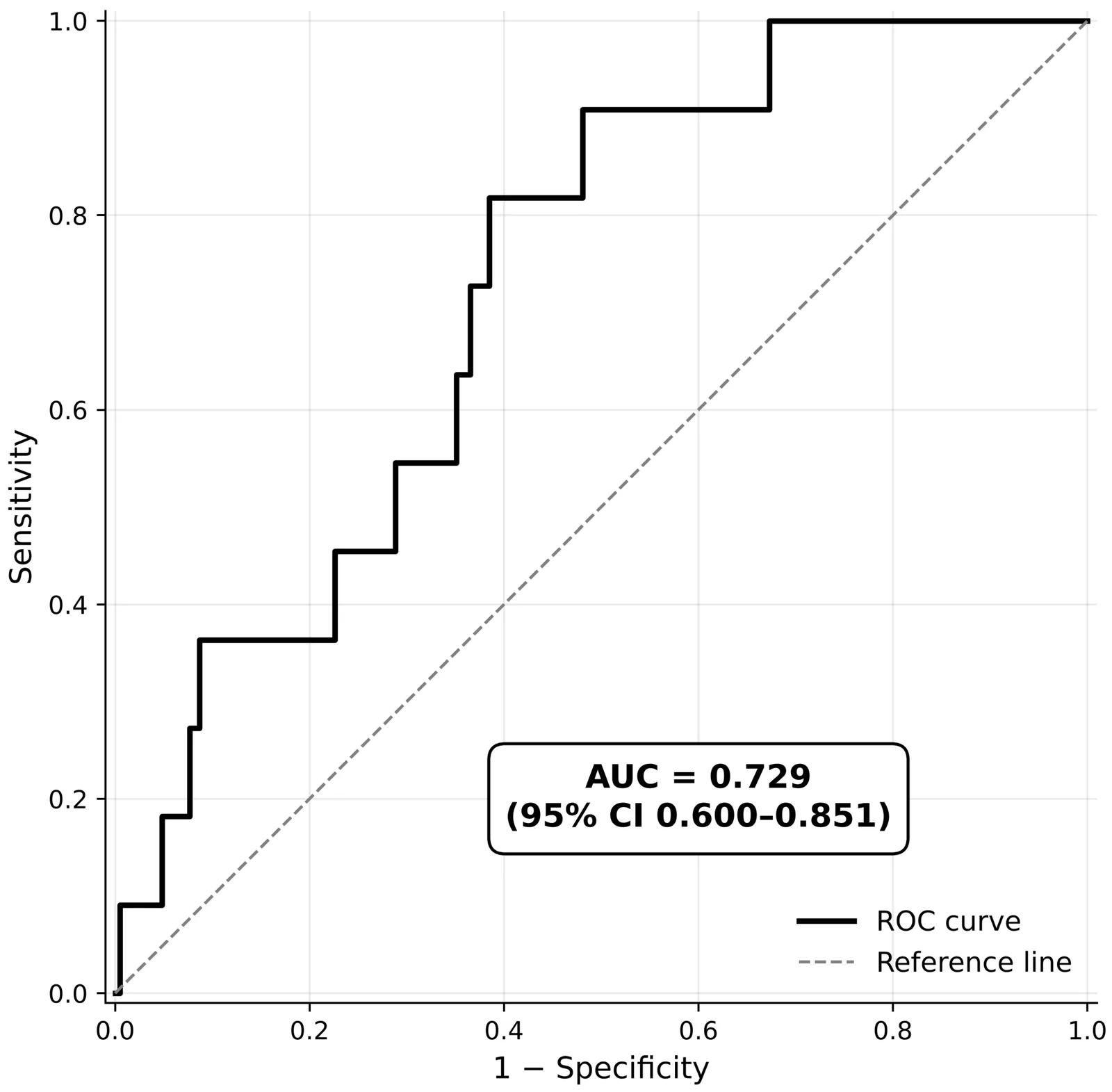
ROC Curve for the Multivariable Model of Cannulation Failure.

For comparison, an equivalent ROC analysis was performed for the multivariable Firth logistic regression model for cannulation failure, in which the CCI was the only independently significant factor (Table 4). The model yielded an AUC of 0.729 (95% bootstrap CI, 0.600–0.851) (Figure 5). Given the small number of cannulation failure events, this ROC analysis was considered exploratory.

## 4. Discussion

In our retrospective cohort study involving 693 ERCP procedures with no prior papillary intervention, we found that atypical papillary morphologies, classified as Small, Bulging, or Ridged, were associated with lower cannulation success, longer cannulation time, more cannulation attempts, and longer hospital stay compared to regular papillary morphology. In contrast, the presence of PAD did not show a significant association with any of the procedural outcomes or complications. In multivariable analyses adjusted for age, sex, ASA class, and comorbidity burden, neither papillary morphology nor diverticulum status was independently associated with difficult cannulation, overall complications, cannulation failure, or PEP. In contrast, the CCI was the only variable independently associated with cannulation failure.

Our findings regarding papillary morphology are broadly consistent with the existing literature. The four-category classification used in our study was structurally similar to the validated Haraldsson classification [10]. In our study, the rate of difficult cannulation increased from 29.6% in regular papillae to 43.2% in small papillae and 50.0% in ridged papillae. This distribution is largely similar to the 26–41% distribution reported according to papilla types in a recent systematic review and meta-analysis evaluating papilla morphology [11]. The broadly similar distribution observed in our routine clinical cohort provides additional support for a potential association between papillary morphology and procedural difficulty.

Notably, the Others (atypical morphology) group had a significantly higher rate of malignant biliary strictures (16.8% vs. 5.2%; *p* = 0.002) and a lower rate of benign strictures (6.7% vs. 15.2%; *p* = 0.040) compared to the regular group. This suggests that the atypical papillary appearance may not only be a feature that mechanically complicates cannulation, but may also be a visible indicator of a different underlying disease distribution more strongly associated with malignancy. Indeed, previous studies have reported that a bulging papillary appearance, in particular, may be associated with malignant pancreatobiliary diseases [25]. When subtypes were evaluated separately, the rate of malignant stricture was found to increase from 5.2% in regular papilla to 15.4% in small papilla, 15.7% in bulging papilla, and 30.0% in ridged papilla (*p* = 0.004; Table 3). This finding points to a stratified distribution, suggesting that the relationship is not limited to a single morphology.

Our findings regarding the relationship between papillary morphology and malignancy are partially consistent with previous literature. In a study evaluating bulging duodenal papillae, it was reported that the final diagnosis was malignancy in 54.4% of patients; irregular papillary morphology, pancreatic duct dilation, and high direct bilirubin levels together showed high performance in differentiating malignancy (AUC = 0.896) [25]. Similarly, abnormal papillary appearances such as enlargement, protrusion, surface irregularity, hardness, fragility, and ulceration have been described in ampullary neoplasms [26]. In our study, the numerically higher rate of malignant biliary strictures not only in bulging papillae but also in small and ridged morphologies compared to regular papillae suggests that atypical papillary appearance may be associated with underlying malignant disease. However, this finding should be interpreted with caution, especially due to the small sample size of the ridged subgroup and the retrospective design of the study.

The higher prevalence of malignant biliary stricture in patients with atypical papillary morphology should also be considered when interpreting the procedural outcomes. Malignant obstruction may itself increase cannulation difficulty through distortion or infiltration of the distal bile duct and papilla, and in our cohort, malignant biliary obstruction was associated with the lowest cannulation success rate and the highest rates of difficult cannulation and advanced/rescue technique use. Therefore, part of the association between atypical morphology and procedural difficulty observed in univariable analyses may reflect the underlying malignant disease rather than papillary morphology itself, and these two factors cannot be fully disentangled in a retrospective cohort of this size.

Although the rates of difficult cannulation differed among the four papillary morphologies, this difference did not reach statistical significance (*p* = 0.150). Difficult cannulation was numerically more frequent in patients with atypical morphology (39.9% vs. 29.6%), although the difference was not statistically significant (*p* = 0.054). In contrast, significant differences were found in terms of cannulation success, cannulation time, and number of cannulation attempts. The inability to obtain statistical significance for difficult cannulation may be related to limited statistical power, especially due to the small sample size (n = 12) of the Ridged subgroup. Indeed, a recent systematic review and meta-analysis showed that although the rates of difficult cannulation varied between 26% and 41% among papillary morphologies, the difference between the groups did not reach statistical significance [11].

The analysis of cannulation techniques provides further insight into the relationship between papillary morphology and procedural difficulty. Although the overall rate of advanced/rescue technique use did not differ significantly between the Regular and Others groups, the distribution of techniques differed: needle-knife precut papillotomy and fistulotomy were used more frequently in patients with atypical papillae, whereas transpancreatic septotomy was used more frequently in patients with regular papillae. This pattern supports previous observations that papillary morphology may influence the selection of the rescue technique in difficult biliary cannulation [3,9]. In addition, advanced/rescue techniques were required in 39.2% of patients with malignant biliary obstruction, in line with the greater cannulation difficulty observed in this group. However, the small number of patients in each technique subgroup does not allow further interpretation of these findings.

Our finding of no significant association between PAD and cannulation outcomes is broadly consistent with contemporary evidence. Although previous meta-analyses have suggested an overall association between PAD and cannulation failure, subgroup analyses restricted to more recent studies have generally failed to demonstrate a significant association, possibly reflecting advances in cannulation techniques and equipment [13,27]. Similarly, in our cohort, PAD was not associated with difficult cannulation or cannulation success. However, our findings regarding complications differ somewhat from previous meta-analytic evidence, which has reported increased risks of PEP and bleeding in patients with PAD; no significant associations with these complications were observed in our cohort [13,27].

However, the relatively small number of patients with PAD (n = 125) and the low number of individual complication events may have limited the precision of these estimates and our ability to detect moderate associations. Therefore, the absence of statistically significant associations should not be interpreted as evidence of no association, and these findings should be interpreted with caution.

The age distribution of our cohort was consistent with previous ERCP series. The mean age of our patients was relatively high (66.9 ± 17.0 years) and comparable to that reported by Zheng et al. in patients undergoing ERCP (67.6 ± 14.7 years) [28]. In line with their findings, in which patients with PAD were older than those without PAD (mean, 71.9 vs. 63.9 years) [28], patients with PAD in our cohort were significantly older than those without PAD (median, 77.0 vs. 69.0 years; *p* = 0.0002; Table 1). In addition, patients with malignant biliary stricture were older than those without malignant stricture (mean, 71.8 vs. 66.4 years), although this difference did not reach statistical significance (*p* = 0.113). This pattern is in agreement with data from the Swedish national GallRiks registry, in which patients undergoing ERCP for malignancy were older than those undergoing ERCP for common bile duct stones (mean, 71.6 vs. 67.1 years) [29].

The presence of PAD was also associated with some differences in the distribution of underlying diseases in our cohort. The rate of malignant biliary stricture was significantly lower in patients with PAD compared to those without PAD (3.6% vs. 12.1%; *p* = 0.015), while the rate of benign stricture was numerically higher, but this difference was not statistically significant (10.9% vs. 7.6%; *p* = 0.341) (Table 1). Our results regarding the higher prevalence of malignant stricture in patients without PAD appear to be consistent with other studies in the literature [30]. These findings suggest that the relationship between PAD and pancreatobiliary disease profile may vary in different patient groups. Indeed, a large single-center study reported higher rates of choledocholithiasis (81.9% vs. 75.0%; *p* < 0.001) and pancreatic head cancer (4.78% vs. 2.32%; *p* = 0.023) in patients with PAD [31]. The relationship between PAD and biliary stone disease has been explained by mechanisms such as diverticulum-related biliary stasis, bacterial colonization, and subsequent stone formation [31,32]. However, the fact that only four cases of malignant biliary stricture were found among patients with PAD in our study significantly limits the precision of this estimate and the interpretation of this finding. When evaluated together with the findings regarding papillary morphology, our results suggest that the anatomical features examined may be related not only to the local mechanics of cannulation but also to the underlying pancreatobiliary disease profile that led to the indication for ERCP. It may be useful to consider these possible differences in case distribution in future studies and to stratify the analyses according to the underlying diagnosis whenever possible.

Considering that classifying PAD solely as present/absent might mask clinically significant heterogeneity, patients with PAD were also classified as Type I, IIa, IIb, III, IVa, and IVb according to the Li-Tanaka classification, which is based on the position of the papilla relative to the diverticulum edge [20]. In our cohort, Type IIb (papilla diverticulum edge, outer side, <1 cm) was the most common subtype (59.2%). No significant differences were found in the results evaluated between subtypes (all *p* > 0.10); however, these estimates should be interpreted with caution due to the fact that the number of patients in some subgroups decreased to n = 5. Interestingly, the direction of our statistically insignificant results differed from the results in the study in which this classification was defined. While the original study reported Type IIa as the most difficult subtype in terms of cannulation (31.4% difficult cannulation) and Type IIb as relatively easy (8.3%) [20], in our cohort, the lowest numerical rate of difficult cannulation was observed in Type IIa (0%; n = 10) and the highest rate in Type IVa (60.0%; n = 5). Due to the low number of patients in both comparisons, we do not consider this difference as a finding that contradicts the original classification study. On the contrary, it shows how variable subtype-level estimates can be without sufficiently large, preferably multicenter samples. Therefore, in studies of this size, it is considered more appropriate to use the PAD presence/absence comparison, which is more powerful for primary analysis, while subtype analyses are used for hypothesis generation purposes.

In our study, comorbidity burden, as measured by the comorbidity-only CCI, was the only variable independently associated with cannulation failure after adjustment for papillary morphology, diverticulum status, age, sex, and ASA class (adjusted OR = 1.40 per one-point increase; 95% CI, 1.03–1.91; *p* = 0.031). This result is conceptually consistent with a previous study showing that CCI was an independent predictor of adverse events after ERCP in patients aged 90 years and older [33]. However, our cohort was not limited to very elderly patients, and the outcome we evaluated was cannulation failure rather than a post-procedural adverse event, thereby extending these observations to a different outcome. The role of standardized comorbidity measures in ERCP-specific risk assessment has been investigated to a more limited extent than anatomical and procedural factors [14,15]. The mechanisms underlying this association cannot be determined from our data. Because the CCI assigns points to solid tumors and metastatic disease, part of this association may reflect underlying malignancy, which was associated with lower cannulation success in our cohort; other factors, such as frailty or reduced tolerance of prolonged procedures and sedation, may also have contributed. Given the small number of cannulation failure events, this finding should be considered hypothesis-generating and requires validation. If confirmed in independent cohorts, future studies could evaluate whether incorporating a standardized comorbidity index alongside ASA classification improves pre-procedural risk stratification.

Some methodological choices in this study are also important. First, we limited our primary analysis to patients with native papillae because previous sphincterotomy alters papillary anatomy, and cannulation characteristics may differ between native and previously treated papillae [34,35]. Second, the use of Firth penalized logistic regression instead of standard maximum likelihood estimation was preferred to address the separation problem arising from the data structure. The combination of small and unbalanced subgroups with low event numbers led to non-finite or unstable coefficient estimates in the Ridged papilla subgroup and, in some models, for PAD presence in standard logistic regression. The Firth method reduces bias and provides finite coefficient estimates in the presence of separation [24].

Overall, our findings offer some clinical implications for ERCP risk assessment. Papillary morphology and PAD are anatomical features that can be directly assessed by the endoscopist at the start of the procedure. Although papillary morphology was associated with some parameters related to cannulation success and procedural difficulty in univariable analyses, neither papillary morphology nor PAD was independently associated with the evaluated outcomes. In contrast, CCI was the only variable independently associated with cannulation failure. These findings suggest that focusing solely on local anatomical features or the presence of PAD may not be sufficient for ERCP risk assessment, and that considering comorbidity burden may provide additional information for pre-procedural risk assessment. However, further validation of this relationship in larger, prospective cohorts is needed to establish the clinical relevance of the association between CCI and cannulation failure.

This study has some important limitations that should be considered. First, papillary morphology was recorded in only 381 (55%) of 693 patients. This situation may have reduced the precision of the analyses regarding papillary morphology, and although no systematic reason for the missing documentation was identified, some degree of selection bias cannot be completely excluded. The reduced complete-case sample size in the multivariable analyses may have further limited statistical power. Furthermore, the number of individual complication events was low, limiting the precision and statistical power of the multivariable analyses for complication outcomes. The low number of events may also have contributed to the inability to demonstrate the complication risks reported in larger meta-analyses as being associated with PAD in our study. Second, the retrospective and single-center design of the study limits the generalizability of the results, and because the primary operator was not documented for each procedure, the potential influence of individual endoscopist experience could not be assessed. Additionally, the fact that the number of patients in some subgroups in the comparisons regarding Li-Tanaka subtypes decreased to n = 5 limits the precision of these analyses; therefore, subtype results should be considered exploratory. Third, the multivariable model for cannulation failure included only 11 events. The low number of events limits the precision of effect estimates and the generalizability of the model’s discriminative performance [36]. Fourth, only the total CCI score was available in our database, and the individual comorbidities contributing to the score were not recorded separately; therefore, we could not compare CCI subcomponents between groups or determine which specific comorbidities drove the observed association between CCI and cannulation failure.

## 5. Conclusions

In this ERCP cohort of patients with native papillae, atypical papillary morphology was associated with lower cannulation success and parameters reflecting greater procedural difficulty. In contrast, no significant association was observed between the presence of periampullary diverticulum and the assessed procedural outcomes, while analyses of PAD subtypes remained exploratory because of small subgroup sizes. Neither papillary morphology nor PAD was independently associated with the evaluated outcomes in multivariable analyses. Among the variables examined, higher Charlson Comorbidity Index was the only factor independently associated with cannulation failure; however, this finding should be interpreted cautiously given the small number of failure events. Larger prospective multicenter studies are needed to validate the potential clinical relevance of papillary morphology and comorbidity burden in ERCP risk assessment.

## Figures and Tables

**Table 1:** Baseline characteristics and procedural/clinical outcomes by periampullary diverticulum status.

Characteristic/Outcome	Diverticulum Absent (n = 508)	Diverticulum Present (n = 125)	*p*
Baseline characteristics			
Age, years, median (IQR)	69.0 (56.0–78.0)	77.0 (67.0–82.0)	0.0002
Female sex, %	57.1	56.0	0.905
Charlson Comorbidity Index, median (IQR)	4.0 (2.0–5.0)	4.0 (3.0–5.0)	0.008
ASA class, median (IQR)	3.0 (2.0–3.0)	3.0 (2.0–4.0)	0.030
Malignant stricture, %	12.1	3.6	0.015
Benign stricture, %	7.6	10.9	0.341
Procedural and clinical outcomes			
Cannulation success, %	96.3	95.2	0.608 ^a^
Difficult cannulation, %	33.9	32.8	0.821
Cannulation time, min, median (IQR)	3.0 (1.0–7.0)	3.0 (2.0–6.0)	0.983 ^b^
Cannulation attempts, median (IQR)	2.0 (1.0–3.0)	2.0 (1.0–3.0)	0.758 ^b^
Overall complications, %	16.8	12.4	0.380
PEP, %	12.2	8.3	0.324
Bleeding, %	1.9	0.0	0.357 ^a^
Perforation, %	0.3	0.0	1.000 ^a^
Cholangitis, %	0.0	0.0	— ^c^
Cardiopulmonary event, %	2.7	4.7	0.319 ^a^
Hospital stay, days, median (IQR)	6.0 (4.0–8.0)	5.0 (4.0–7.0)	0.460 ^b^

IQR, interquartile range; ASA, American Society of Anesthesiologists physical status classification; PEP, post-ERCP pancreatitis. ^a^ Fisher’s exact test; ^b^ Mann–Whitney U test; ^c^ zero events in both groups precluded formal testing; all other *p*-values from chi-square test (categorical) or Mann–Whitney U test (continuous). Complication-related rows (complications, PEP, bleeding, perforation, cholangitis, cardiopulmonary events, hospital stay) were ascertained in a subset of each group due to incomplete complication documentation in the source records; percentages are calculated over patients with the field recorded, not over the full column-header totals.

**Table 2:** Baseline characteristics and procedural and clinical outcomes according to papilla morphology: Regular versus Others.

Characteristic/Outcome	Regular (n = 234)	Others (n = 147)	*p*
Baseline characteristics			
Age, years, median (IQR)	70.0 (58.0–79.0)	71.0 (57.5–81.0)	0.509
Female sex, %	54.7	57.1	0.718
Charlson Comorbidity Index, median (IQR)	4.0 (2.5–5.0)	4.0 (2.8–6.0)	0.231
ASA class, median (IQR)	3.0 (2.0–3.0)	3.0 (2.0–3.0)	0.600
Malignant stricture, %	5.2	16.8	0.002
Benign stricture, %	15.2	6.7	0.040
Procedural and clinical outcomes			
Cannulation success, %	97.4	91.2	0.012
Difficult cannulation, %	29.6	39.9	0.054
Cannulation time, min, median (IQR)	3.0 (1.0–5.0)	4.0 (1.8–8.0)	0.009 ^a^
Cannulation attempts, median (IQR)	2.0 (1.0–3.0)	2.0 (1.0–3.0)	0.007 ^a^
Overall complications, %	9.3	7.3	0.745
PEP, %	7.3	4.2	0.467
Bleeding, %	2.0	2.1	1.000 ^b^
Perforation, %	0.0	0.0	— ^c^
Cholangitis, %	0.0	0.0	— ^c^
Cardiopulmonary event, %	0.7	1.0	1.000 ^b^
Hospital stay, days, median (IQR)	5.0 (4.0–7.0)	6.0 (4.0–9.0)	0.013 ^a^

PEP, post-ERCP pancreatitis. ^a^ Mann–Whitney U test; ^b^ Fisher’s exact test; ^c^ zero events precluded formal testing; all other *p*-values from chi-square test. Complications, PEP, bleeding, cardiopulmonary events, and hospital stay were ascertained in a subset of each group (approximately 65% of Regular, 66% of Others for complications; approximately 56% for hospital stay) due to incomplete complication documentation in the source records; percentages are calculated over patients with the field recorded. Cannulation success, difficulty, time, and attempts were available for nearly the full group.

**Table 3:** Disease characteristics and procedural and clinical outcomes according to the four papilla morphology categories.

Outcome	Regular (n = 234)	Small (n = 46)	Bulging (n = 89)	Ridged (n = 12)	*p*
Disease characteristics					
Malignant stricture, %	5.2	15.4	15.7	30.0	0.004 ^a^
Benign stricture, %	15.2	7.7	5.7	10.0	0.158 ^a^
Procedural and clinical outcomes					
Cannulation success, %	97.4	89.1	92.1	91.7	0.044 ^a^
Difficult cannulation, %	29.6	43.2	36.8	50.0	0.150
Cannulation time, min, median (IQR)	3.0 (1.0–5.0)	3.0 (1.0–7.0)	4.0 (2.0–8.0)	4.0 (3.5–9.5)	0.020 ^b^
Cannulation attempts, median (IQR)	2.0 (1.0–3.0)	2.0 (1.0–3.0)	2.0 (1.0–3.0)	3.0 (2.0–5.0)	0.005 ^b^
Overall complications, %	9.3	10.0	5.2	12.5	0.752 ^a^
PEP, %	7.3	6.7	3.4	0.0	0.654 ^a^
Bleeding, %	2.0	3.3	1.7	0.0	0.929 ^a^
Perforation, %	0.0	0.0	0.0	0.0	— ^c^
Cholangitis, %	0.0	0.0	0.0	0.0	— ^c^
Cardiopulmonary event, %	0.7	0.0	0.0	12.5	0.002 ^a,d^
Hospital stay, days, median (IQR)	5.0 (4.0–7.0)	6.0 (5.0–10.0)	6.0 (4.0–8.0)	8.0 (6.0–8.5)	0.034 ^b^

PEP, post-ERCP pancreatitis; IQR, interquartile range. Data are presented as percentages for categorical variables and median (IQR) for continuous variables. ^a^ Chi-square test or Fisher’s exact test, as appropriate according to expected cell counts; ^b^ Kruskal–Wallis test; ^c^ no events were observed in any group, precluding formal statistical testing; ^d^ nominally significant result based on only two cardiopulmonary events among 247 patients and should therefore be interpreted with caution. Malignant and benign stricture analyses were based on the 310 patients with both papilla morphology and stricture status available. Post-ERCP complications (overall complications, PEP, bleeding, and cardiopulmonary events) and length of hospital stay were available only for subsets of patients because of incomplete documentation in the source records. Accordingly, percentages were calculated using the number of patients with available data for the respective outcome as the denominator. Cannulation success, difficult cannulation, cannulation time, and number of cannulation attempts were available for nearly the entire papilla morphology cohort.

**Table 4:** Multivariable Firth penalized logistic regression analysis of cannulation failure.

Predictor	Adjusted OR (95% CI)	*p*
Papilla: Small vs. Regular	2.00 (0.44–9.15)	0.371
Papilla: Bulging vs. Regular	1.22 (0.31–4.75)	0.776
Papilla: Ridged vs. Regular	1.59 (0.05–48.10)	0.791
Diverticulum: Present vs. Absent	1.63 (0.44–6.12)	0.466
Sex: Male vs. Female	0.94 (0.31–2.87)	0.912
Age, per year	1.01 (0.97–1.06)	0.537
ASA physical status class, per unit	0.54 (0.24–1.17)	0.118
CCI (comorbidity-only), per point	1.40 (1.03–1.91) *	0.031 *

Reference categories: papilla morphology = Regular; PAD = Absent; sex = Female. ASA (American Society of Anesthesiologists). CCI (comorbidity-only), Charlson Comorbidity Index after subtraction of the age points. Estimates were obtained using Firth penalized logistic regression in the primary cohort of patients with native papillae (n = 219; 11 cannulation failure events). Firth penalization was used because of quasi-complete separation under standard maximum-likelihood estimation. Statistically significant results are indicated by an asterisk (* *p* < 0.05).

## Data Availability

The datasets generated and analyzed during the current study are available from the corresponding author on reasonable request.
